# The genome sequence of the common earthworm, *Lumbricus terrestris *(Linnaeus, 1758)

**DOI:** 10.12688/wellcomeopenres.20178.1

**Published:** 2023-10-30

**Authors:** Mark L. Blaxter, David Spurgeon, Peter Kille, Wellcome Sanger Institute Tree of Life programme, Wellcome Sanger Institute Scientific Operations: DNA Pipelines collective, Tree of Life Core Informatics collective, Darwin Tree of Life Consortium

**Affiliations:** 1 Tree of Life, Wellcome Sanger Institute, Hinxton, England, UK; 2 UK Centre for Ecology & Hydrology, Wallingford, England, UK; 3 Cardiff School of Biosciences, Cardiff University, Cardiff, Wales, UK

**Keywords:** Lumbricus terrestris, common earthworm, genome sequence, chromosomal, Haplotaxida

## Abstract

We present a genome assembly from an individual *Lumbricus terrestris* (the common earthworm; Annelida; Clitellata; Haplotaxida; Lumbricidae). The genome sequence is 1,056.5 megabases in span. Most of the assembly is scaffolded into 18 chromosomal pseudomolecules. The mitochondrial genome has also been assembled and is 15.93 kilobases in length.

## Species taxonomy

Eukaryota; Metazoa; Eumetazoa; Bilateria; Protostomia; Spiralia; Lophotrochozoa; Annelida; Clitellata; Oligochaeta; Crassiclitellata; Lumbricina; Lumbricidae; Lumbricinae; *Lumbricus*; *Lumbricus terrestris* complex; *Lumbricus terrestris* (Linnaeus, 1758) (NCBI:txid6398).

## Background

The Darwin Tree of Life project aims to sequence to reference quality the genomes of species of Ireland and Britain ( Blaxter *et al.*, 2022). While most species we target make their homes on these islands, some are ecosystem engineers that shape the environments in which they and others live. One such group is the lumbricid annelids, or earthworms, which collectively generate and maintain soils by processing and degrading mineral particles, cycling biological material into the soil, aerating and draining soil through their burrows and providing rich habitats for soil microbes in their casts ( Kooch & Jalilvand, 2008). The importance of earthworms as not just inhabitants but prime generators of soil was noted by Charles Darwin in his last book “ *The Formation of Vegetable Mould Through the Action of Worms, with Observations on their Habits*” ( Darwin, 1881).

*Lumbricus terrestris*, the common earthworm (see ( NBN Atlas Partnership, 2023) was described by Linnaeus in his “Systema Naturae” in 1758 (genus #246, on pages 647–648 in a paraphyletic “Vermes”) ( Linnaeus, 1758). Now placed in Lumbricidae within the oligochaete annelids (phylum Annelida, class Oligochaeta, order Crassiclitellata), *L. terrestris* is the largest of the earthworms of Ireland and Britain, and has, since Darwin, been the focus of intensive study especially in the areas of ecotoxicology and ecotoxicogenomics ( Brulle *et al.*, 2010; Sheppard, 1998; Stürzenbaum *et al.*, 2009). As *L. terrestris*, like other earthworms, carries out respiratory exchange through its skin, and consumes and processes soil matter through its gut, it is particularly exposed to geogenic and anthropogenic contamination of soils ( Stürzenbaum *et al.*, 2004). Here we present a chromosomally-complete assembly of *L. terrestris*, collected from the Wellcome Sanger Institute campus, to support ongoing work on this keystone species.

## Genome sequence report

**Figure 1.**
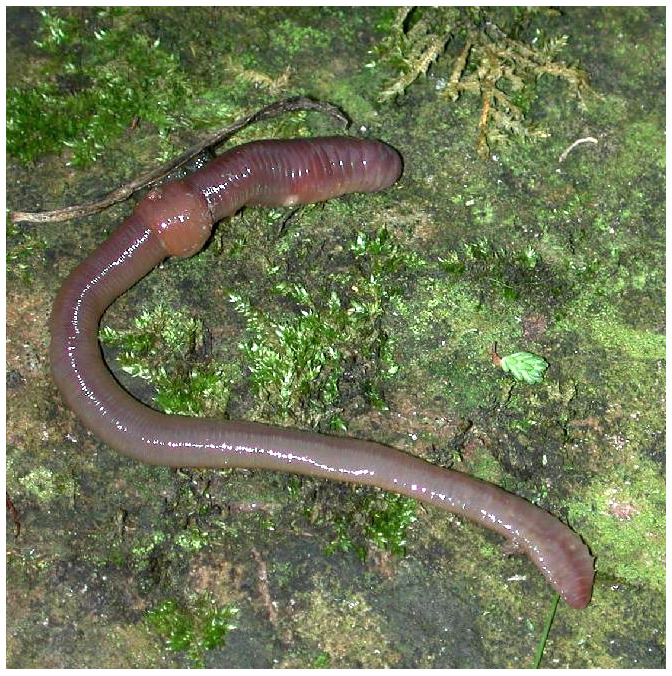
Photograph of *Lumbricus terrestris* by Michael Linnenbach.

The genome was sequenced from one *Lumbricus terrestris* ( Figure 1) collected from Wellcome Genome Campus, Hinxton, UK (52.08, 0.18). We generated 33-fold coverage using Pacific Biosciences single-molecule HiFi long reads. Primary assembly contigs were scaffolded with chromosome conformation Hi-C data. Manual assembly curation corrected 199 missing joins or mis-joins and removed 221 haplotypic duplications, reducing the assembly length by 11.9% and the scaffold number by 33.33%, and decreasing the scaffold N50 by 4.45%.

**Figure 2.**
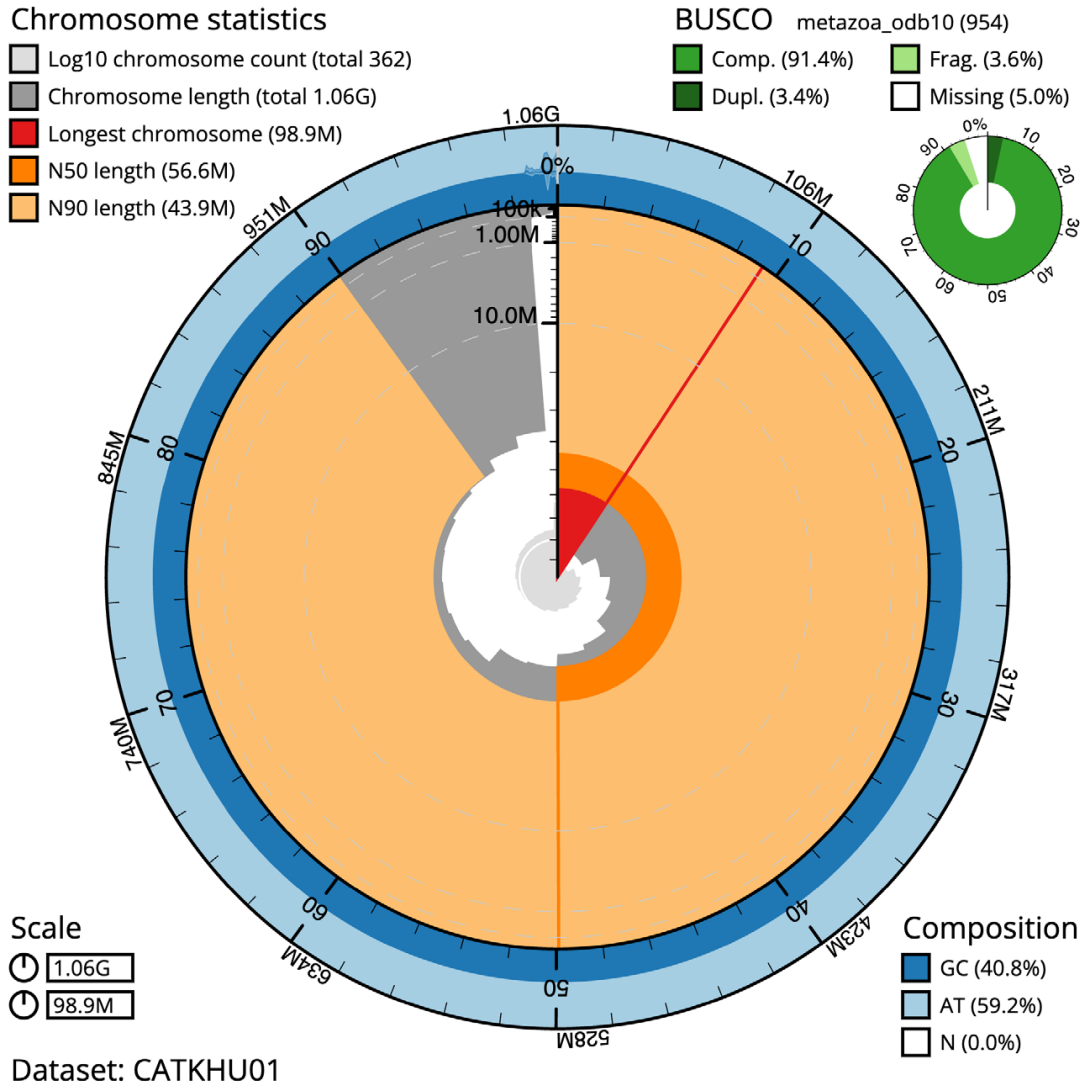
Genome assembly of *Lumbricus terrestris*, wcLumTerr1.1: metrics. The BlobToolKit Snailplot shows N50 metrics and BUSCO gene completeness. The main plot is divided into 1,000 size-ordered bins around the circumference with each bin representing 0.1% of the 1,056,547,181 bp assembly. The distribution of scaffold lengths is shown in dark grey with the plot radius scaled to the longest scaffold present in the assembly (98,895,420 bp, shown in red). Orange and pale-orange arcs show the N50 and N90 scaffold lengths (56,588,617 and 43,887,615 bp), respectively. The pale grey spiral shows the cumulative scaffold count on a log scale with white scale lines showing successive orders of magnitude. The blue and pale-blue area around the outside of the plot shows the distribution of GC, AT and N percentages in the same bins as the inner plot. A summary of complete, fragmented, duplicated and missing BUSCO genes in the metazoa_odb10 set is shown in the top right. An interactive version of this figure is available at https://blobtoolkit.genomehubs.org/view/wcLumTerr1.1/dataset/CATKHU01/snail.

**Figure 3.**
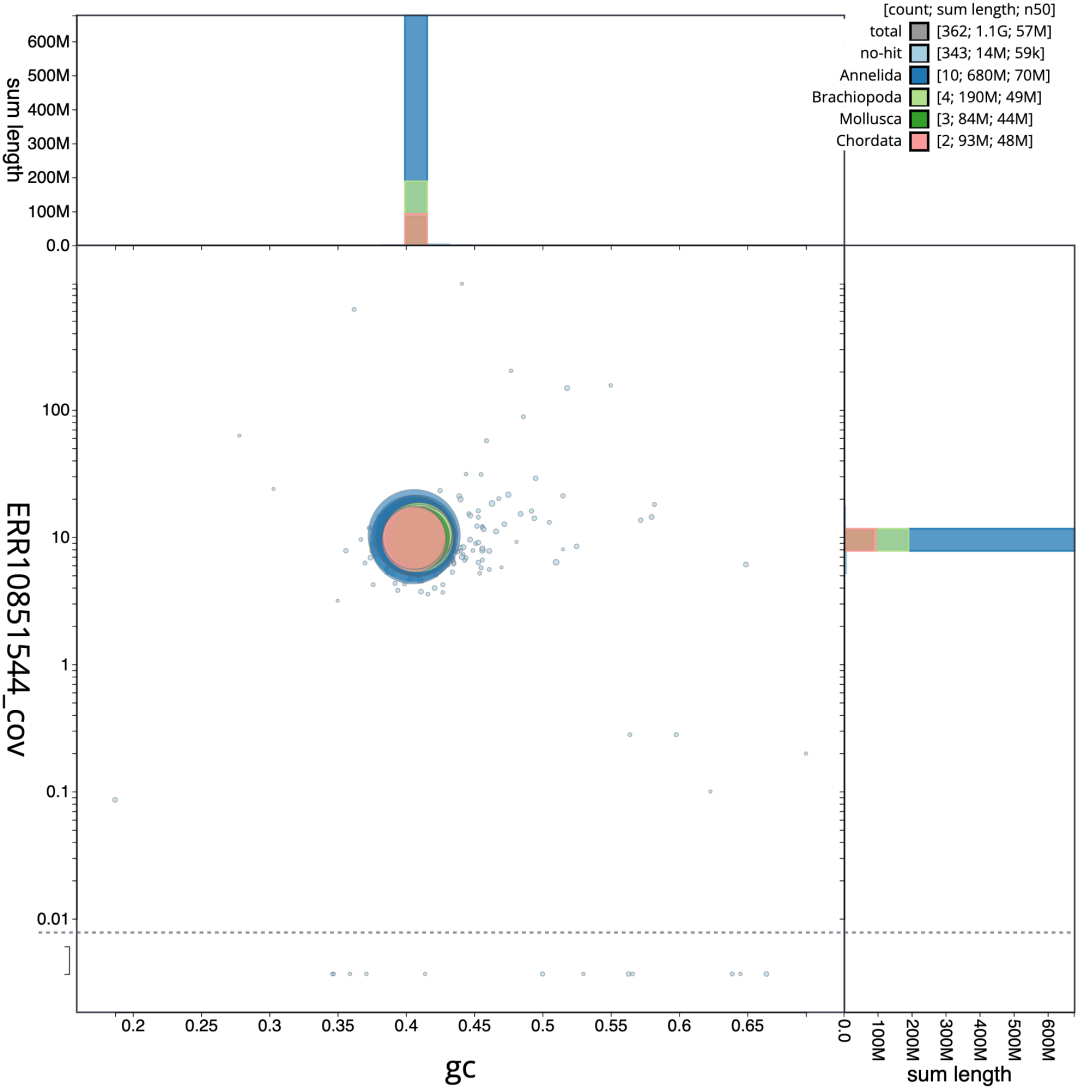
Genome assembly of *Lumbricus terrestris*, wcLumTerr1.1: BlobToolKit GC-coverage plot. Scaffolds are coloured by phylum. Circles are sized in proportion to scaffold length. Histograms show the distribution of scaffold length sum along each axis. An interactive version of this figure is available at https://blobtoolkit.genomehubs.org/view/wcLumTerr1.1/dataset/CATKHU01/blob.

**Figure 4.**
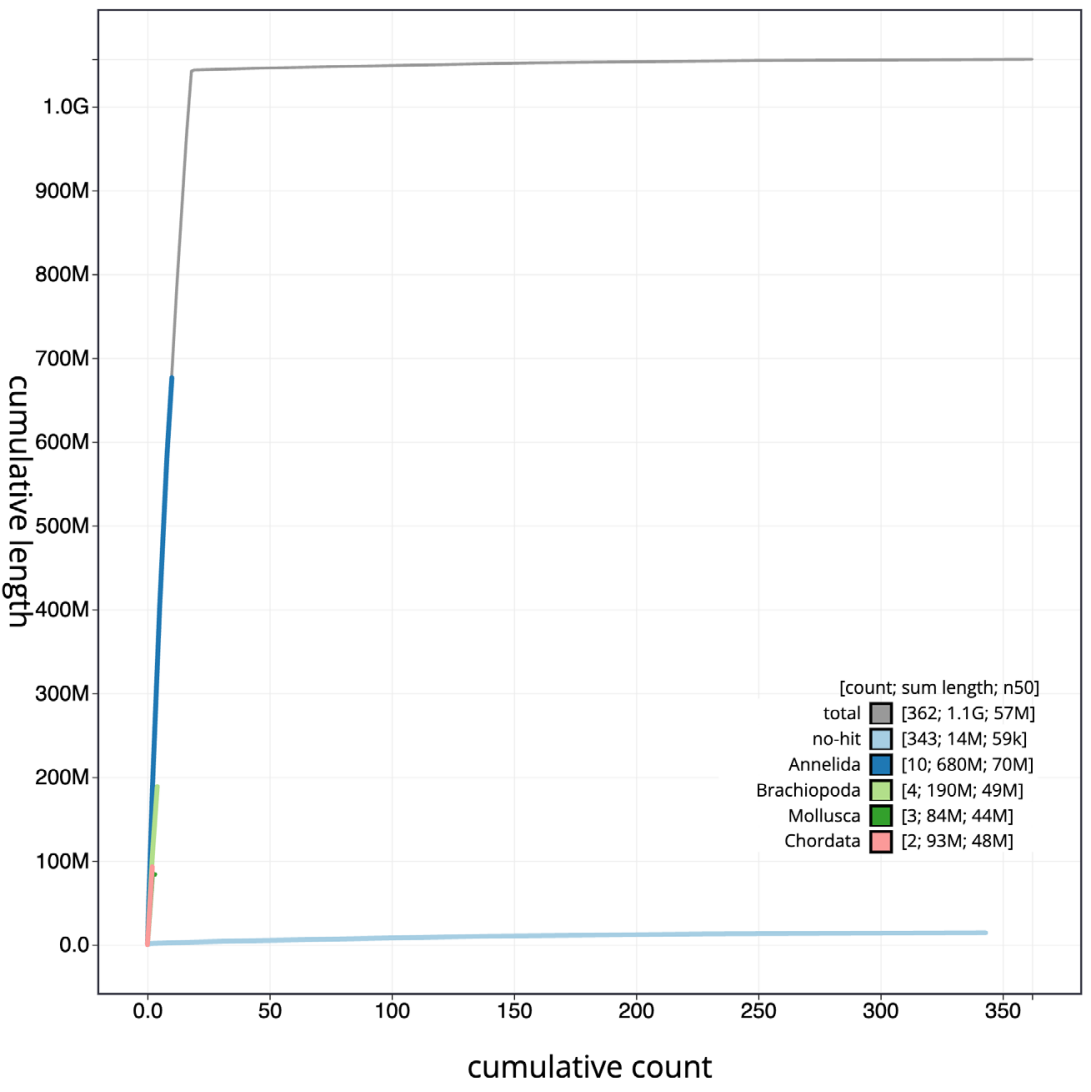
Genome assembly of *Lumbricus terrestris*, wcLumTerr1.1: BlobToolKit cumulative sequence plot. The grey line shows cumulative length for all scaffolds. Coloured lines show cumulative lengths of scaffolds assigned to each phylum using the buscogenes taxrule. An interactive version of this figure is available at https://blobtoolkit.genomehubs.org/view/wcLumTerr1.1/dataset/CATKHU01/cumulative.

**Figure 5.**
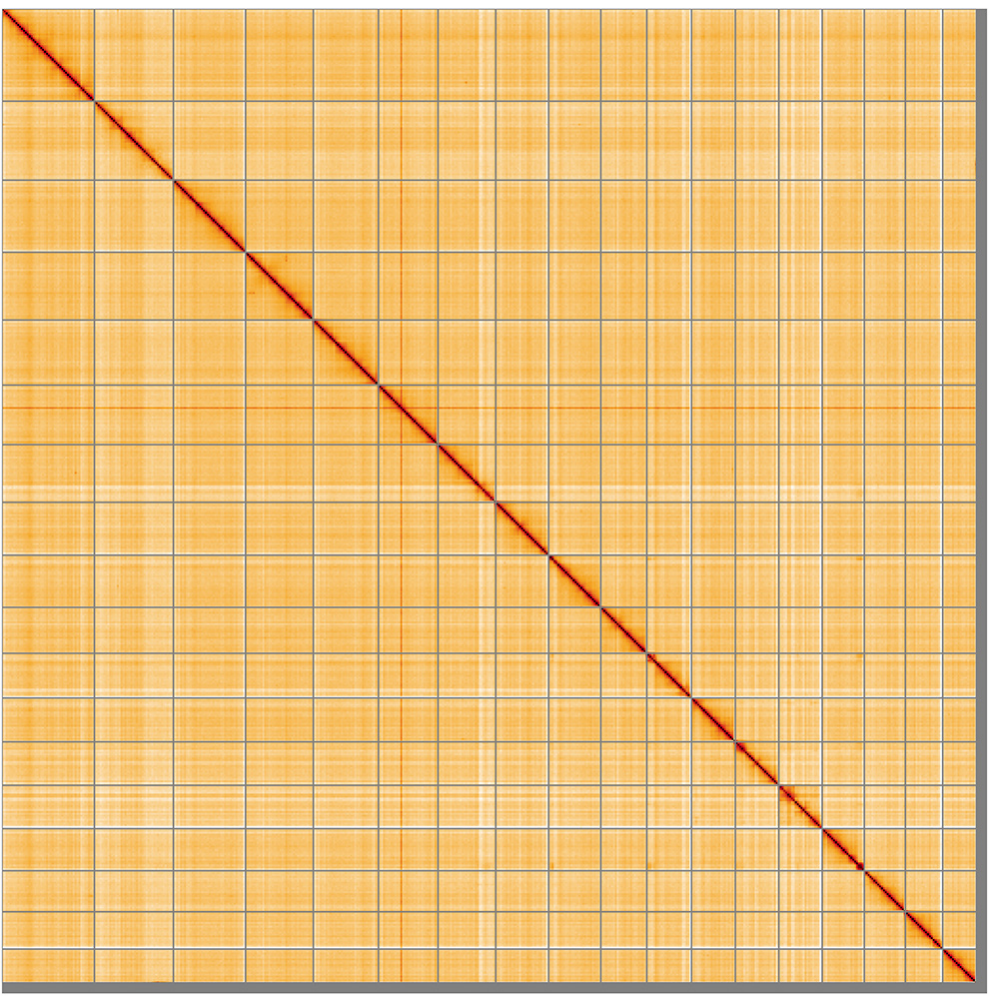
Genome assembly of *Lumbricus terrestris*, wcLumTerr1.1: Hi-C contact map of the wcLumTerr1.1 assembly, visualised using HiGlass. Chromosomes are shown in order of size from left to right and top to bottom. An interactive version of this figure may be viewed at https://genome-note-higlass.tol.sanger.ac.uk/l/?d=OfTjARz6QZKy250DRKimLg.

The final assembly has a total length of 1056.5 Mb in 361 sequence scaffolds with a scaffold N50 of 56.6 Mb ( Table 1). The snailplot in Figure 2 provides a summary of the assembly statistics, while the distribution of assembly scaffolds on GC proportion and coverage is shown in Figure 3. The cumulative assembly plot in Figure 4 shows curves for subsets of scaffolds assigned to different phyla. Most (98.77%) of the assembly sequence was assigned to 18 chromosomal-level scaffolds. Chromosome-scale scaffolds confirmed by the Hi-C data are named in order of size ( Figure 5; Table 2). While not fully phased, the assembly deposited is of one haplotype. Contigs corresponding to the second haplotype have also been deposited. The mitochondrial genome was also assembled and can be found as a contig within the multifasta file of the genome submission.

**Table 1.** Genome data for *Lumbricus terrestris*, wcLumTerr1.1.

Project accession data		
Assembly identifier	wcLumTerr1.1	
Species	*Lumbricus terrestris*	
Specimen	wcLumTerr1	
NCBI taxonomy ID	6398	
BioProject	PRJEB59400	
BioSample ID	SAMEA7524126	
Isolate information	wcLumTerr1: bodywall and intestine (DNA sequencing and Hi-C scaffolding) wcLumTerr2: bodywall (RNA sequencing)	
Assembly metrics *		*Benchmark*
Consensus quality (QV)	57.3	*≥ 50*
*k*-mer completeness	99.99%	*≥ 95%*
BUSCO **	C:91.4%[S:88.1%,D:3.4%], F:3.6%,M:5.0%,n:954	*C ≥ 95%*
Percentage of assembly mapped to chromosomes	98.77%	*≥ 95%*
Sex chromosomes	-	*localised homologous pairs*
Organelles	Mitochondrial genome assembled	*complete single alleles*
Raw data accessions		
PacificBiosciences SEQUEL II	ERR10841333, ERR10841334, ERR10841335	
Hi-C Illumina	ERR10851548, ERR10851550	
PolyA RNA-Seq Illumina	ERR10851549	
Genome assembly		
Assembly accession	GCA_949752735.1	
*Accession of alternate haplotype*	GCA_949752785.1	
Span (Mb)	1056.5	
Number of contigs	1587	
Contig N50 length (Mb)	1.6	
Number of scaffolds	361	
Scaffold N50 length (Mb)	56.6	
Longest scaffold (Mb)	98.9	

* Assembly metric benchmarks are adapted from column VGP-2020 of “Table 1: Proposed standards and metrics for defining genome assembly quality” from ( Rhie *et al.*, 2021). ** BUSCO scores based on the metazoa_odb10 BUSCO set using v5.3.2. C = complete [S = single copy, D = duplicated], F = fragmented, M = missing, n = number of orthologues in comparison. A full set of BUSCO scores is available at https://blobtoolkit.genomehubs.org/view/wcLumTerr1.1/dataset/CATKHU01/busco.

**Table 2.** Chromosomal pseudomolecules in the genome assembly of *Lumbricus terrestris*, wcLumTerr1.

INSDC accession	Chromosome	Length (Mb)	GC%
OX457036.1	1	98.9	40.5
OX457037.1	2	84.43	40.5
OX457038.1	3	77.17	41.0
OX457039.1	4	72.47	40.5
OX457040.1	5	69.63	40.5
OX457041.1	6	63.79	41.0
OX457042.1	7	61.74	41.0
OX457043.1	8	56.59	41.0
OX457044.1	9	55.79	41.0
OX457045.1	10	49.13	41.0
OX457046.1	11	47.79	40.5
OX457047.1	12	46.9	41.0
OX457048.1	13	46.6	41.0
OX457049.1	14	46.28	40.5
OX457050.1	15	45.14	40.5
OX457051.1	16	43.89	41.0
OX457052.1	17	39.89	41.0
OX457053.1	18	36.07	41.5
OX457054.1	MT	0.02	36.0

The estimated Quality Value (QV) of the final assembly is 57.3 with *k*-mer completeness of 99.99%, and the assembly has a BUSCO v5.3.2 completeness of 91.4% (single = 88.1%, duplicated = 3.4%), using the metazoa_odb10 reference set ( *n* = 954).

Metadata for specimens, spectral estimates, sequencing runs, contaminants and pre-curation assembly statistics can be found at https://links.tol.sanger.ac.uk/species/6398.

## Methods

### Sample acquisition and nucleic acid extraction

*Lumbricus terrestris* specimens were collected from Wellcome Genome Campus (latitude 52.08, longitude 0.18) on 2020-03-12. The specimen was taken from soil in woodland by turning leaflitter and subsoil. The collectors were Mark Blaxter (Wellcome Sanger Institute), Peter Kille (University of Cardiff) and David Spurgeon (UK Centre for Ecology & Hydrology) and David Spurgeon identified the specimen. The specimen was flash frozen in liquid nitrogen. One specimen was used for DNA sequencing and Hi-C data (specimen ID SAN0001206, ToLID wcLumTerr1) and another specimen (specimen ID SAN0001207, ToLID wcLumTerr2) was used for RNA sequencing.

DNA was extracted at the Tree of Life laboratory, Wellcome Sanger Institute (WSI). The wcLumTerr1 sample was weighed and dissected on dry ice with tissue set aside for Hi-C sequencing. Bodywall and intestinal tissue was disrupted using a Nippi Powermasher fitted with a BioMasher pestle. High molecular weight (HMW) DNA was extracted using the Qiagen MagAttract HMW DNA extraction kit. HMW DNA was sheared into an average fragment size of 12–20 kb in a Megaruptor 3 system with speed setting 30. Sheared DNA was purified by solid-phase reversible immobilisation using AMPure PB beads with a 1.8X ratio of beads to sample to remove the shorter fragments and concentrate the DNA sample. The concentration of the sheared and purified DNA was assessed using a Nanodrop spectrophotometer and Qubit Fluorometer and Qubit dsDNA High Sensitivity Assay kit. Fragment size distribution was evaluated by running the sample on the FemtoPulse system.

RNA was extracted from tissue of wcLumTerr2 in the Tree of Life Laboratory at the WSI using TRIzol, according to the manufacturer’s instructions. RNA was then eluted in 50 μl RNAse-free water and its concentration assessed using a Nanodrop spectrophotometer and Qubit Fluorometer using the Qubit RNA Broad-Range (BR) Assay kit. Analysis of the integrity of the RNA was done using Agilent RNA 6000 Pico Kit and Eukaryotic Total RNA assay.

### Sequencing

Pacific Biosciences HiFi circular consensus DNA sequencing libraries were constructed according to the manufacturers’ instructions. Poly(A) RNA-Seq libraries were constructed using the NEB Ultra II RNA Library Prep kit. DNA and RNA sequencing was performed by the Scientific Operations core at the WSI on Pacific Biosciences SEQUEL II (HiFi) and Illumina HiSeq 4000 (RNA-Seq) instruments. Hi-C data were also generated from body wall and intestinal tissue of wcLumTerr1 using the Arima2 kit and sequenced on the HiSeq X Ten and Illumina NovaSeq 6000 instrument.

### Genome assembly, curation and evaluation

Assembly was carried out with Hifiasm ( Cheng *et al.*, 2021) and haplotypic duplication was identified and removed with purge_dups ( Guan *et al.*, 2020). The assembly was then scaffolded with Hi-C data ( Rao *et al.*, 2014) using YaHS ( Zhou *et al.*, 2023). The assembly was checked for contamination and corrected as described previously ( Howe *et al.*, 2021). Manual curation was performed using HiGlass ( Kerpedjiev *et al.*, 2018) and Pretext ( Harry, 2022). The mitochondrial genome was assembled using MitoHiFi ( Uliano-Silva *et al.*, 2023), which runs MitoFinder ( Allio *et al.*, 2020) or MITOS ( Bernt *et al.*, 2013) and uses these annotations to select the final mitochondrial contig and to ensure the general quality of the sequence.

A Hi-C map for the final assembly was produced using bwa-mem2 ( Vasimuddin *et al.*, 2019) in the Cooler file format ( Abdennur & Mirny, 2020). To assess the assembly metrics, the *k*-mer completeness and QV consensus quality values were calculated in Merqury ( Rhie *et al.*, 2020). This work was done using Nextflow ( Di Tommaso *et al.*, 2017) DSL2 pipelines “sanger-tol/readmapping” ( Surana *et al.*, 2023a) and “sanger-tol/genomenote” ( Surana *et al.*, 2023b). The genome was analysed within the BlobToolKit environment ( Challis *et al.*, 2020) and BUSCO scores ( Manni *et al.*, 2021; Simão *et al.*, 2015) were calculated.

Table 3 contains a list of relevant software tool versions and sources.

**Table 3.** Software tools: versions and sources.

Software tool	Version	Source
BlobToolKit	4.1.7	https://github.com/blobtoolkit/blobtoolkit
BUSCO	5.3.2	https://gitlab.com/ezlab/busco
Hifiasm	0.16.1-r375	https://github.com/chhylp123/hifiasm
HiGlass	1.11.6	https://github.com/higlass/higlass
Merqury	MerquryFK	https://github.com/thegenemyers/MERQURY.FK
MitoHiFi	2	https://github.com/marcelauliano/MitoHiFi
PretextView	0.2	https://github.com/wtsi-hpag/PretextView
purge_dups	1.2.3	https://github.com/dfguan/purge_dups
sanger-tol/genomenote	v1.0	https://github.com/sanger-tol/genomenote
sanger-tol/readmapping	1.1.0	https://github.com/sanger-tol/readmapping/tree/1.1.0
YaHS	1.2a	https://github.com/c-zhou/yahs

### Wellcome Sanger Institute – Legal and Governance

The materials that have contributed to this genome note have been supplied by a Darwin Tree of Life Partner. The submission of materials by a Darwin Tree of Life Partner is subject to the **‘Darwin Tree of Life Project Sampling Code of Practice’**, which can be found in full on the Darwin Tree of Life website here. By agreeing with and signing up to the Sampling Code of Practice, the Darwin Tree of Life Partner agrees they will meet the legal and ethical requirements and standards set out within this document in respect of all samples acquired for, and supplied to, the Darwin Tree of Life Project. 

Further, the Wellcome Sanger Institute employs a process whereby due diligence is carried out proportionate to the nature of the materials themselves, and the circumstances under which they have been/are to be collected and provided for use. The purpose of this is to address and mitigate any potential legal and/or ethical implications of receipt and use of the materials as part of the research project, and to ensure that in doing so we align with best practice wherever possible. The overarching areas of consideration are:

• Ethical review of provenance and sourcing of the material

• Legality of collection, transfer and use (national and international) 

Each transfer of samples is further undertaken according to a Research Collaboration Agreement or Material Transfer Agreement entered into by the Darwin Tree of Life Partner, Genome Research Limited (operating as the Wellcome Sanger Institute), and in some circumstances other Darwin Tree of Life collaborators.

## Data Availability

European Nucleotide Archive: *Lumbricus terrestris* (common earthworm). Accession number PRJEB59400; https://identifiers.org/ena.embl/PRJEB59400. ( Wellcome Sanger Institute, 2023) The genome sequence is released openly for reuse. The *Lumbricus terrestris* genome sequencing initiative is part of the Darwin Tree of Life (DToL) project. All raw sequence data and the assembly have been deposited in INSDC databases. The genome will be annotated using available RNA-Seq data and presented through the Ensembl pipeline at the European Bioinformatics Institute. Raw data and assembly accession identifiers are reported in Table 1.

## Author information

Members of the Wellcome Sanger Institute Tree of Life programme are listed here: https://doi.org/10.5281/zenodo.4783585.

Members of Wellcome Sanger Institute Scientific Operations: DNA Pipelines collective are listed here: https://doi.org/10.5281/zenodo.4790455.

Members of the Tree of Life Core Informatics collective are listed here: https://doi.org/10.5281/zenodo.5013541.

Members of the Darwin Tree of Life Consortium are listed here: https://doi.org/10.5281/zenodo.4783558.

## References

[ref-1] Abdennur N Mirny LA : Cooler: Scalable storage for Hi-C data and other genomically labeled arrays. *Bioinformatics.* 2020;36(1):311–316. 10.1093/bioinformatics/btz540 31290943 PMC8205516

[ref-2] Allio R Schomaker‐Bastos A Romiguier J : MitoFinder: Efficient automated large‐scale extraction of mitogenomic data in target enrichment phylogenomics. *Mol Ecol Resour.* 2020;20(4):892–905. 10.1111/1755-0998.13160 32243090 PMC7497042

[ref-3] Bernt M Donath A Jühling F : MITOS: Improved *de novo* metazoan mitochondrial genome annotation. *Mol Phylogenet Evol.* 2013;69(2):313–319. 10.1016/j.ympev.2012.08.023 22982435

[ref-4] Blaxter M Mieszkowska N Di Palma F : Sequence locally, think globally: The Darwin Tree of Life Project. *Proc Natl Acad Sci U S A.* 2022;119(4): e2115642118. 10.1073/pnas.2115642118 35042805 PMC8797607

[ref-5] Brulle F Morgan AJ Cocquerelle C : Transcriptomic underpinning of toxicant-mediated physiological function alterations in three terrestrial invertebrate taxa: A review. *Environ Pollut.* 2010;158(9):2793–2808. 10.1016/j.envpol.2010.06.019 20619942

[ref-6] Challis R Richards E Rajan J : BlobToolKit - interactive quality assessment of genome assemblies. *G3 (Bethesda).* 2020;10(4):1361–1374. 10.1534/g3.119.400908 32071071 PMC7144090

[ref-7] Cheng H Concepcion GT Feng X : Haplotype-resolved *de novo* assembly using phased assembly graphs with hifiasm. *Nat Methods.* 2021;18(2):170–175. 10.1038/s41592-020-01056-5 33526886 PMC7961889

[ref-9] Darwin C : The Formation of Vegetable Mould through the Action of Worms, with Observations on Their Habits.London, UK: John Murray,1881. Reference Source

[ref-10] Di Tommaso P Chatzou M Floden EW : Nextflow enables reproducible computational workflows. *Nat Biotechnol.* 2017;35(4):316–319. 10.1038/nbt.3820 28398311

[ref-13] Guan D McCarthy SA Wood J : Identifying and removing haplotypic duplication in primary genome assemblies. *Bioinformatics.* 2020;36(9):2896–2898. 10.1093/bioinformatics/btaa025 31971576 PMC7203741

[ref-14] Harry E : PretextView (Paired REad TEXTure Viewer): A desktop application for viewing pretext contact maps. 2022; [Accessed 19 October 2022]. Reference Source

[ref-15] Howe K Chow W Collins J : Significantly improving the quality of genome assemblies through curation. *GigaScience.* Oxford University Press,2021;10(1): giaa153. 10.1093/gigascience/giaa153 33420778 PMC7794651

[ref-16] Kerpedjiev P Abdennur N Lekschas F : HiGlass: web-based visual exploration and analysis of genome interaction maps. *Genome Biol.* 2018;19(1): 125. 10.1186/s13059-018-1486-1 30143029 PMC6109259

[ref-17] Kooch Y Jalilvand H : Earthworms as Ecosystem Engineers and the Most Important Detritivors in Forest Soils. *Pak J Biol Sci.* 2008;11(6):819–825. 10.3923/pjbs.2008.819.825 18814642

[ref-18] Linnaeus C : Systema Naturae.10th ed. Stockholm: Holmiae Salvius,1758.

[ref-19] Manni M Berkeley MR Seppey M : BUSCO update: Novel and streamlined workflows along with broader and deeper phylogenetic coverage for scoring of eukaryotic, prokaryotic, and viral genomes. *Mol Biol Evol.* 2021;38(10):4647–4654. 10.1093/molbev/msab199 34320186 PMC8476166

[ref-20] NBN Atlas Partnership: *Lumbricus terrestris* Linnaeus, 1758 Common Earthworm. *NBN Atlas.* 2023; [Accessed 9 September 2023]. Reference Source

[ref-21] Rao SSP Huntley MH Durand NC : A 3D map of the human genome at kilobase resolution reveals principles of chromatin looping. *Cell.* 2014;159(7):1665–1680. 10.1016/j.cell.2014.11.021 25497547 PMC5635824

[ref-22] Rhie A McCarthy SA Fedrigo O : Towards complete and error-free genome assemblies of all vertebrate species. *Nature.* 2021;592(7856):737–746. 10.1038/s41586-021-03451-0 33911273 PMC8081667

[ref-23] Rhie A Walenz BP Koren S : Merqury: Reference-free quality, completeness, and phasing assessment for genome assemblies. *Genome Biol.* 2020;21(1): 245. 10.1186/s13059-020-02134-9 32928274 PMC7488777

[ref-24] Sheppard S : Advances in Earthworm Ecotoxicology: Proceedings from the Second International Workshop on Earthworm Ecotoxicology, April 1997, Amsterdam, the Netherlands.Setac Press,1998. Reference Source

[ref-25] Simão FA Waterhouse RM Ioannidis P : BUSCO: assessing genome assembly and annotation completeness with single-copy orthologs. *Bioinformatics.* 2015;31(19):3210–3212. 10.1093/bioinformatics/btv351 26059717

[ref-26] Stürzenbaum SR Andre J Kille P : Earthworm genomes, genes and proteins: the (re)discovery of Darwin’s worms. *Proc Biol Sci.* 2009;276(1658):789–797. 10.1098/rspb.2008.1510 19129111 PMC2664377

[ref-27] Stürzenbaum SR Georgiev O Morgan AJ : Cadmium Detoxification in Earthworms: From Genes to Cells. *Environ Sci Technol.* 2004;38(23):6283–6289. 10.1021/es049822c 15597883

[ref-28] Surana P Muffato M Qi G : sanger-tol/readmapping: sanger-tol/readmapping v1.1.0 - Hebridean Black (1.1.0). *Zenodo.* 2023a; [Accessed 21 July 2023]. 10.5281/zenodo.7755665

[ref-29] Surana P Muffato M Sadasivan Baby C : sanger-tol/genomenote (v1.0.dev). *Zenodo.* 2023b; [Accessed 21 July 2023]. 10.5281/zenodo.6785935

[ref-30] Uliano-Silva M Ferreira JGRN Krasheninnikova K : MitoHiFi: a python pipeline for mitochondrial genome assembly from PacBio high fidelity reads. *BMC Bioinformatics.* 2023;24(1): 288. 10.1186/s12859-023-05385-y PMC10354987 37464285

[ref-31] Vasimuddin M Misra S Li H : Efficient Architecture-Aware Acceleration of BWA-MEM for Multicore Systems.In: *2019 IEEE International Parallel and Distributed Processing Symposium (IPDPS).*IEEE,2019;314–324. 10.1109/IPDPS.2019.00041

[ref-33] Wellcome Sanger Institute: The genome sequence of the common earthworm, *Lumbricus terrestris* (Linnaeus, 1758). European Nucleotide Archive.[dataset], accession number PRJEB59400,2023. 10.12688/wellcomeopenres.20178.1 PMC10799228 38249959

[ref-32] Zhou C McCarthy SA Durbin R : YaHS: yet another Hi-C scaffolding tool. *Bioinformatics.* 2023;39(1): btac808. 10.1093/bioinformatics/btac808 36525368 PMC9848053

