# Supplementary material for: The genome sequence of the common earthworm, Lumbricus terrestris (Linnaeus, 1758)
Source: Wellcome Open Res. 2023 Oct 30;8:500. [Version 1] doi: 10.12688/wellcomeopenres.20178.1 (PMC10799228; doi:10.12688/wellcomeopenres.20178.1)
Supplement: Peer review history

# Reviewer report: Reviewer response for version 1

**DOI:** 10.21956/wellcomeopenres.22341.r71542

**Published:** 2024-01-25

**Authors:** José María Martín-Durán

**Affiliations:** 1 School of Biological and Behavioural Sciences, Queen Mary University of London, London, UK

This genome note reports the assembly of the common earthworm *Lumbricus terrestris*. This is a broadly distributed segmented worm, very familiar to the public. The assembly meets the high standards of the Darwin Tree of Life initiative and will be an essential resource to a diverse range of disciplines, from developmental biology to ecology, ecotoxicology and conservation.

Are sufficient details of methods and materials provided to allow replication by others?

Yes

Is the rationale for creating the dataset(s) clearly described?

Yes

Are the datasets clearly presented in a useable and accessible format?

Yes

Are the protocols appropriate and is the work technically sound?

Yes

Reviewer Expertise:

Evolutionary biology, comparative genomics, evo-devo, annelids

I confirm that I have read this submission and believe that I have an appropriate level of expertise to confirm that it is of an acceptable scientific standard.

# Reviewer report: Reviewer response for version 1

**DOI:** 10.21956/wellcomeopenres.22341.r71560

**Published:** 2024-01-19

**Authors:** Sudhakar Sivasubramaniam, Arun Arumugaperumal

**Affiliations:** 1 Department of Biotechnology, Manonmaniam Sundaranar University, Tirunelveli, Tamil Nadu, India; 2 Department of Biotechnology, Rajalakshmi Engineering College, Chennai, Tamilnadu, India

The authors report the genome sequence of Lumbricus terrestris earthworm. The genome size was 1056.5 Mbp and the sequences were assembled in to 18 chromosomes.The genome completeness analysis showed that the assembly was 91.4% complete.

 The authors have used best performing softwares for the bioinformatics work. They could have gone an extra mile to predict the non-coding genes present in the genome. It would have been an interesting data to look in to the exon-intron junctions and gene structure.

Are sufficient details of methods and materials provided to allow replication by others?

Yes

Is the rationale for creating the dataset(s) clearly described?

Yes

Are the datasets clearly presented in a useable and accessible format?

Yes

Are the protocols appropriate and is the work technically sound?

Yes

Reviewer Expertise:

Regeneration and stem cell biology, Genomics and Transcriptomics

We confirm that we have read this submission and believe that we have an appropriate level of expertise to confirm that it is of an acceptable scientific standard.

# Reviewer report: Reviewer response for version 1

**DOI:** 10.21956/wellcomeopenres.22341.r71561

**Published:** 2024-01-19

**Authors:** Yoshiaki Morino

**Affiliations:** 1 Institute of Life and Environmental Sciences, University of Tsukuba, Tsukuba, Japan

The paper reports genome sequencing of the common earthworm, Lumbricus terrestris. The chromosome-level assembly was generated using standard methods and is sufficiently high quality. The significance of the species is well introduced. I have only two minor comments:

1. Concerning the absence of sex chromosomes, it would be beneficial to include a mention in the manuscript that earthworms, including this species, are generally hermaphrodites.
2. The manuscript should describe the source tissues used for the RNA-seq.

Are sufficient details of methods and materials provided to allow replication by others?

Yes

Is the rationale for creating the dataset(s) clearly described?

Yes

Are the datasets clearly presented in a useable and accessible format?

Yes

Are the protocols appropriate and is the work technically sound?

Yes

Reviewer Expertise:

Marine invertebrate, Evo-Devo

I confirm that I have read this submission and believe that I have an appropriate level of expertise to confirm that it is of an acceptable scientific standard.
